# Mitoribosomal Deregulation Drives Senescence via TPP1-Mediated Telomere Deprotection

**DOI:** 10.3390/cells11132079

**Published:** 2022-06-30

**Authors:** Seongki Min, So Mee Kwon, Jiwon Hong, Young-Kyoung Lee, Tae Jun Park, Su Bin Lim, Gyesoon Yoon

**Affiliations:** 1 Department of Biochemistry, Ajou University School of Medicine, Suwon 16499, Korea; tjdrl4100@nate.com (S.M.); hjw2784@naver.com (J.H.); algamsa109@naver.com (Y.-K.L.); park64@ajou.ac.kr (T.J.P.); 2 Department of Biomedical Sciences, Graduate School, Ajou University, Suwon 16499, Korea; som137kwon@gmail.com; 3 Department of Physiology, Ajou University School of Medicine, Suwon 16499, Korea

**Keywords:** mitoribosome, replicative senescence, shelterin, telomere maintenance

## Abstract

While mitochondrial bioenergetic deregulation has long been implicated in cellular senescence, its mechanistic involvement remains unclear. By leveraging diverse mitochondria-related gene expression profiles derived from two different cellular senescence models of human diploid fibroblasts, we found that the expression of mitoribosomal proteins (MRPs) was generally decreased during the early-to-middle transition prior to the exhibition of noticeable SA-β-gal activity. Suppressed expression patterns of the identified senescence-associated MRP signatures (SA-MRPs) were validated in aged human cells and rat and mouse skin tissues and in aging mouse fibroblasts at single-cell resolution. TIN2- and POT1-interaction protein (TPP1) was concurrently suppressed, which induced senescence, accompanied by telomere DNA damage. Lastly, we show that SA-MRP deregulation could be a potential upstream regulator of TPP1 suppression. Our results indicate that mitoribosomal deregulation could represent an early event initiating mitochondrial dysfunction and serve as a primary driver of cellular senescence and an upstream regulator of shelterin-mediated telomere deprotection.

## 1. Introduction

Mitochondrial oxidative phosphorylation (OXPHOS)-mediated bioenergetic deregulation has long been implicated in cellular senescence and the organismal aging process, supporting the mitochondrial free radical theory of aging [1,2,3]. A mitochondrion possesses its own DNA and the independent genetic information transfer systems, such as replication, transcription, and translation machineries, which are collectively termed mitochondrial central dogma (MCD) systems [4,5]. The nuclear DNA (ncDNA)-encoded core acting proteins involved in MCD activities are fundamentally different from central dogma systems of ncDNA origin [6]. Mammalian mitochondrial DNA (mtDNA), which has a length of about 16 kb circular double-stranded DNA, is highly vulnerable to oxidative stress due to its nucleoid structure without chromatin-like packing and its proximity to reactive oxygen species (ROS)-generating OXPHOS sites [7]. The accumulation of somatic mutations and/or deletions in mtDNA is considered to be an important contributor to senescence and the aging process [8,9], which is further evidenced by the study of DNA polymerase γ-deficient mice [10]. Nevertheless, it remains unclear whether mtDNA damage is the only cause of senescence-associated mitochondrial dysfunction and how the MCD activity is involved in the dysfunction. There are only 13 mtDNA-encoded proteins synthesized by MCD activity, although these protein products are essential components of the OXPHOS system governing aerobic ATP production [11]. The mitochondrial ribosome (mitoribosome), the final executer of MCD, is a specialized macrostructure that executes the translation of the 13 proteins. Structurally, the mitoribosome is formed by 82 ncDNA-encoded mitoribosomal proteins (MRPs) and two mtDNA-encoded ribosomal RNAs (rRNAs) [12], indicating that well-orchestrated expression of the 82 MRPS and 2 rRNAs is critical to modulate mitochondrial bioenergetic activity.

After a finite number of population doublings, primary human diploid fibroblasts (HDFs) enter a state, termed replicative senescence (RS), in which their growth is irreversibly arrested [13]. RS has been employed as a well-established in vitro cell aging model to investigate the normal process of organismal aging [14]. The most extensively studied stimuli leading to RS is telomere attrition caused by the “end-replication problem” during DNA replication [15,16]. It suppresses mitochondrial biogenesis through proliferator-activated receptor γ coactivator (PGC)-1α and/or PGC-1β through p53, mTOR, or PARP activation [17], generating a telomere-to-mitochondria axis to accelerate the aging process. On the other hand, the telomeric G triplet is particularly sensitive to cleavage upon oxidative damage [18,19], implying the contribution of mitochondrial deregulation-induced ROS to telomere attrition. Human telomeres are assembled with a characteristic multiprotein complex, named shelterin, which is comprised of numerous copies of six proteins: telomere-related factor 1 (TRF1), TRF2, repressor/activator protein (RAP1), protection of telomeres protein 1 (POT1), TRF1-interacting nuclear protein 2 (TIN2), and TIN2- and POT1-interaction protein (TPP1) [20,21]. Shelterin protects telomere sequences through modulating both telomeric DNA structures and telomerase activity [17]. p53-dependent TRF2 degradation [22] and human antigen R (HuR) loss-mediated TIN2 destabilization [23] were reported to contribute to the induction and maintenance of cellular senescence. These findings suggest that altered expressions or activities of shelterin factors regulate cellular senescence via telomere deprotection-mediated damage.

Senescent cells accumulate in mammalian tissues with aging [24] at different rates and in an asynchronous manner. We previously reported that HDFs during RS progressively undergo four stages of senescence with distinct gene expression profiles [25], suggesting their gradual, and not abrupt, progress into senescence. Corroborating our findings from the RS model, multi-omics analysis of human epidermal tissues revealed four different phases of aging progression [26], implying that the proportions of cells at different stages may be linked to certain phases of aging progression. In this study, through comprehensive analysis of time-series transcriptomes of RS [25], oxidative stress-induced senescence (OS) of HDF [27], and several other publicly available databases of aged human and mouse cell/tissues transcriptomes, we show that mitoribosomal deregulation could serve as a primary driver of cellular senescence and an upstream regulator of shelterin-mediated telomere deprotection. We further employed bulk and single-cell transcriptomic analytical approaches to investigate whether the cell-to-cell variation and the association of the expression of MRPs and shelterin genes would differ across distinct subpopulations of aging dermal fibroblasts.

## 2. Materials and Methods

### 2.1. Transcriptomic Analysis of Cellular Senescence Models

Processed transcriptome data derived from senescence models (RS and OS) of human primary fibroblasts, previously generated by our group [25,27], were downloaded from the NCBI Gene Expression Omnibus (GEO) under the accession codes GSE41714 and GSE80322, respectively. Probe IDs were mapped to gene symbols using the *mapIds* function in the R *AnnotationDbi* package (v1.52.0). Probes with maximum mean expression levels across samples were collapsed to the genes for subsequent analyses. Principal component analysis (PCA) was performed using the *prcomp* function in the R stats package (v4.0.3). Fuzzy c-means clustering was applied to the scaled data with the pre-determined number of clusters (*n* = 10 for RS; *n* = 4 for OS) using the *mfuzz* function in the R *Mfuzz* package (v2.50.0).

### 2.2. MitoCarta3.0 and Genes Encoding MRPs and RPs

**Figure 1:**
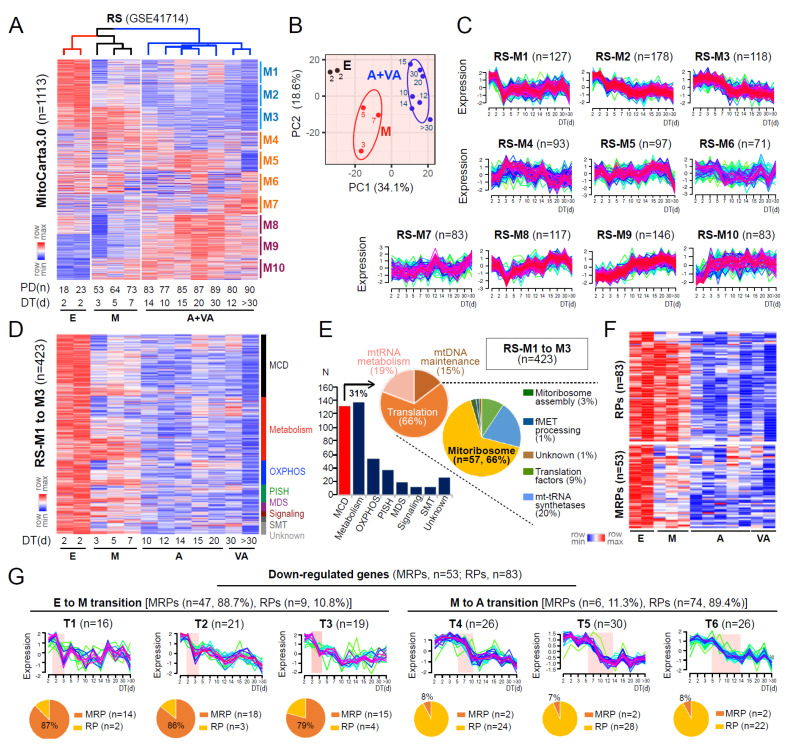
MRP deregulation is a key event occurring during the initial E-to-M transition of replicative senescence. (**A**) Expression heatmap and unsupervised hierarchical clustering (metric = one minus Pearson correleation, linkage method = average) of genes from MitoCarta3.0 in RS of HDFs. DT and PT refer to the doubling time (days) and the number of population doublings to reach the cell condition, respectively. E, M, A, and VA refer to cells at early, middle, advanced, and very advanced stages of senescence, respectively. (**B**) Principal components (PC) plot of RS showing a clear separation between samples at different stages of senescence. (**C**) Expression patterns of M1 to M10 gene clusters identified by fuzzy c-means clustering over DT. Ellipses represent one standard deviation away from the mean of the Gaussian fitted to samples. *n* refers to the number of genes assigned in each cluster. (**D**) Expression heatmap of RS-M1 to M3 gene clusters. MCD = mitochondrial central dogma; OXPHOS = oxidative phosphorylation; PISH = protein import, sorting and homeostasis; MDS = mitochondrial dynamics and surveillance; SMT = small molecule transport. (**E**) Bar chart and pie charts showing the number and proportion of genes assigned to each category defined by MitoCarta3.0. (**F**) Expression heatmap and (**G**) expression patterns of cytosolic ribosomal proteins (RPs, *n* = 83) and mitochondrial ribosomal proteins (MRPs, *n* = 53) showing progressive downregulation over DT, as identified by fuzzy c-means clustering. Pie charts (bottom) depict the proportion of MRP or RP genes present in each gene cluster.

Human gene annotations for the mitochondrial proteome, including sub-mitochondrial compartments and pathways, were obtained directly from MitoCarta3.0 (https://www.broadinstitute.org/mitocarta/mitocarta30-inventory-mammalian-mitochondrial-proteins-and-pathways, last accessed on 5 April 2021) [5]. Of the 1136 genes listed in the MitoCarta3.0, transcriptome data for 1113 genes were available in the Illumina HumanHT-12 v4.0 expression beadchip (GPL10558), a profiling platform used to generate the two datasets. For the fuzzy c-means clustering of the RS model using the genes encoding MRPs (Figure 1F,G and Supplementary Figure S1), the list of genes encoding proteins of the small (*n* = 30) and large (*n* = 52) ribosomal subunit [28] were obtained from the Ban Lab (https://bangroup.ethz.ch/research/nomenclature-of-ribosomal-proteins.html, last accessed on 5 April 2021). The list of genes encoding cytosolic ribosomal proteins (RPs) was obtained directly from the HUGO Gene Nomenclature Committee (HGNC) resource (https://www.genenames.org/data/genegroup/#!/group/646, last accessed on 5 April 2021).

### 2.3. Transcriptomic Analysis of Aging Skin from Mice and Humans

Reads per kilobase of transcript per million mapped reads (RPKM) values derived from mouse skin at five different ages were downloaded directly from the NCBI GEO under the accession code GSE75192 [29]. Processed expression matrices of dermal fibroblasts isolated from young (*n* = 4, 2-month-old) and old (*n* = 4, 18-month-old) mice (GSE110978) were obtained using the *getGeneExpressionFromGEO* function in the R *geneExpressionFromGEO* package (v0.6). For human transcriptome data, quartile normalized expression data for primary dermal fibroblasts from young (*n* = 5, median age = 24) and old (*n* = 5, median age = 70.4) Caucasian males were downloaded directly from the NCBI GEO under the accession codes GSE131938 [30]. Morpheus (https://software.broadinstitute.org/morpheus/, last accessed on 5 April 2022) was used to generate expression heatmaps of MRPs and to hierarchically cluster them using one minus Pearson correlation distance and the average linkage to determine downregulated MRPs in aging across the three analyzed datasets (Supplementary Figure S2). Human gene symbols were converted to mouse symbols using the R *biomaRt* package (v2.46.3).

### 2.4. Single-Cell RNA-Seq Analysis of Murine Aging Dermal Fibroblasts 

Processed expression matrices with raw read counts per cell index of dermal fibroblasts obtained from mice at 3 different ages (1.5/2.5 days old (newborn), two months old (young), and 18 months old (old)) were downloaded from NCBI GEO under the accession code GSE111136 [31]. The R *Seurat* package (v4.0.1) was used to perform normalization, feature selection, scaling of data, PCA, cell clustering, non-linear dimensional reduction (e.g., uniform manifold approximation and projection (UMAP) plotting), and heatmap generation. Briefly, the most variable genes (*n* = 3000) were used to perform PCA on the scaled data, in which the first 10 PCs were used as inputs for cell clustering. A cell cluster predominantly expressing mitochondrial genes was excluded for further analyses (Supplementary Figure S3). Cells of C3 and C4 clusters were extracted to create independent Seurat objects, which were used to identify features differentially expressed (DE) in fibroblasts of young and old mice using the *FindAllMarkers* function. Using the top 100 DE genes (Supplementary Figure S4), gene ontology (GO) enrichment analysis was performed using the *enrichGO* function in the R *clusterProfiler* package (v3.18.1). 

### 2.5. Cell Culture, Cell Growth, and Development of Cellular Senescence

Primary HDFs isolated from the foreskin of a five-year-old boy were provided by Dr. Lim IK (Ajou University) [32] and were cultured in DMEM supplemented with 10% FBS (Gibco, Grand Island, NY, USA) and antibiotics (Antibiotic-Antimycotic™, Gibco, Grand Island, NY, USA) at 37 °C in a humidified incubator with 5% CO_2_, as previously described [25]. Cell growth and viability were monitored by trypan blue staining. At the end point of each experiment, cells were harvested by trypsinization and counted using the Countess™ automated cell counter (Invitrogen, Carlsbad, CA, USA) after staining with 0.4% (*w*/*v*) trypan blue (Invitrogen) to exclude dead cells.

To develop RS, confluent HDFs were continuously subcultured by being transferred evenly into two new dishes, and the numbers of population doublings (PDs) as well as the doubling times (DTs) were continuously monitored, as described previously [25]. The obtained DT values included the adaptation time after seeding HDF onto new plates. To generate OS of HDFs, primary HDFs (DT2) were exposed to H_2_O_2_, as previously described [27].

### 2.6. Skin Tissues from Aging Animals

Young (4-month-old, *n* = 3) and aged (12- and 18-month-old, *n* = 3 each) male C57BL/6 mice were purchased from KBSI (Korea Basic Science Institute, Deajeon, Korea). After sacrifice, the skin tissue was collected and stored at −80 °C. All animal experiments were approved by the institutional animal research ethics committee at Ajou University Medical Center (approval number: 2020-0051). Skin tissues of young (6-month-old, *n* = 3) and aged (15- and 24-month-old, *n* = 3 each) Sprague Dawley (SD) rats were provided by the Aging Tissue Bank of Pusan, Korea.

### 2.7. Determination of Mitochondrial ROS Level and Monitoring of Cell Size and Cell Granularity

To determine mitochondrial ROS level, cells were incubated in media containing 10 μM Mitosox fluorescence probe (Invitrogen) for 15 min at 37 °C. Stained cells were washed and resuspended in PBS and analyzed by flow cytometry (FACS Vantage, Becton Dickinson Corp., San Antonio, TX, USA). Mean values of arbitrary fluorescence units of 10,000 cells were obtained.

### 2.8. SA-β-Gal Activity Staining

Cells seeded on twelve-well plates were subjected to SA-β-gal activity staining. Gain of SA-β-gal activity was monitored by using the SA-β-gal staining kit (CST, #9860S, Danvers, MA, USA) according to the instructions provided. Representative images from each well were taken using a Leica DMi1 inverted microscope (Leica Microsystems, Wetzlar, Germany). By counting the numbers of the blue-stained and total cells using Image J software (National Institutes of Health, https://imagej.nih.gov/ij/, last accessed on 20 May 2022), the percentage of SA-β-gal positive (+) cells was obtained. Three wells were used for each experimental condition and two independent experiments were performed (total *n* = 6).

### 2.9. Transfection of siRNAs and Expression Plasmid into Cells

HDFs were transfected with siRNA duplexes for the target genes using Lipofectamine RNAiMAX™ reagent (Invitrogen, Carlsbad, CA, USA) according to the instructions provided. Target siRNA duplexes were generated by Bioneer (Daejeon, Korea) and their sequences were as follows: MRPS9 (#1, 5′-GAGGUUUCGAAGAAGUGUA; #2, 5′-GAAACUUACACAGAGGAUU), MRPS15 (#1, 5′-CACUUGGAGAAACAUCGAA; #2, 5-GUCUGUCAAGAUCCGCAGU), MRPS31 (#1, 5′-CUCGGCGAUGUUUCCUAGA; #2, 5′-GAUUCUCUCCCUUUUGAUA), ACD (#1, 5′-GACCUAUCUCUGACCCUCA; #2, 5′-CCUACCGGUACCACAGGAA), POT1 (#1, 5′-CUGAUGUGGAUCAACUGAA; #2, 5′-CUGAUCAAGCUACAGUGUA), and negative control (5′-CCUACGCCACCAAUUUGGU). The TPP1 overexpression plasmid (pCMV6-ACD-AC-GFP) was purchased from OriGene Technologies (Rockville, MD, USA) and introduced into HDFs using Fugene^®^ HD Reagent (Promega, Fitchburg, WI, USA). 

### 2.10. Western Blot Analysis

Cells were washed twice with PBS and lysed in RIPA lysis buffer (150 mM NaCl, 1% NP-40, 0.5% Sodium deoxycholate, 0.1% SDS, 50 mM Tris (pH 8.0), 1 mM NaF, 1 mM Na_3_VO_4_, 1 mM PMSF, 1.5 μg/mL pepstatin A, 1.5 μg/mL leupeptin). Antibodies for MRPS9 (GTX87450), MRPS18C (GTX87389), MRPS22 (GTX45001), MRPS30 (GTX54320), MRPL9 (GTX116779), MRPL46 (GTX87311), MT-CYB (GTX45884), and Telomerase (GTX30410) were purchased from GeneTex Inc. (Irvine, CA, USA). Antibodies for MRPS15 (ab137070), MRPS31 (ab167406), MRPL30 (ab179819), and POT1 (ab124784) from Abcam (Cambridge, MA, USA). Antibodies for MRPL11 (2199S) and TPP1 (14667S) were obtained from Cell Signaling Technology (Danvers, MA, USA), and antibodies for NDUFA9 (A21344), ATP5A1 (A21350), SDHA (A11142), and MT-ND6 (A31857) from Invitrogen (Carlsbad, CA, USA). Antibodies for p21 (sc-6246) and β-Actin (sc-8432) were purchased from Santa Cruz (Dallas, TX, USA). β-Actin was used as a loading control to obtain the immuno-blots for diverse target proteins using the same cell lysates. Several blots were cut into two or three pieces for hybridization with different target antibodies and, if necessary, they were re-probed for reconfirmation. The representative blots shown in the results were selected from the results for which actin levels were comparable to each other. For each target protein, at least three independent experiments were performed. Quantitative values of target protein levels on the immune blot were obtained by densitometric analysis using Image J (National Institutes of Health, https://imagej.nih.gov/ij/, last accessed on 20 May 2022). 

### 2.11. Quantitative Real-Time PCR (qPCR) for mRNA Expression

Total cellular RNAs were isolated using an RNA isolation plus kit (MN, Dueren, Germany), and their cDNAs were prepared using ReverTra Ace™ qPCR RT Master Mix (Toyobo Co. Ltd., Osaka, Japan). PCR was performed using GoTaq^®^ qPCR Master Mix (Promega, Fitchburg, WI, USA) according to the protocol provided. The primer sets for targets were produced by Macrogen (Seoul, Korea). 

To isolate total RNAs from animal skin tissues, the Maxwell RSC Simply RNA Tissue kit (Promega, Fitchburg, WI, USA) was used according to the manufacturer’s protocol. Sequences of the primer sets used are listed in Supplementary Table S1.

### 2.12. Estimation of Telomere Length

Telomere length was estimated by qPCR against genomic DNA as described previously, with a slight modification [33]. Total cellular genomic DNA was isolated using the conventional method. To estimate telomere length, qPCR was performed using GoTaq^®^ qPCR Master Mix (Promega, Fitchburg, WI, USA). The primer sets were produced by Macrogen (Seoul, Korea). Acidic ribosomal phosphoprotein P0 (36B4) gene, a known single copy gene, was used as an internal control. Sequences of the primer sets used are as follows: telomere, 5′-CGGTTTGTTTGGGTTTGGGTTTGGGTTTGGGTTTGGGTT and 5′-GGCTTGCCTTACCCTTACCCTTACCCTTACCCTTACCCT; 36B4, 5′-CAGCAAGTGGGAAGGTGTAATCC and 5′-CCCATTCTATCATCAACGGGTACAA.

### 2.13. Monitoring Mitochondrial Translation Activity

Mitochondrial translation activity was monitored using a Click-iTTM HPG Alexa FluorTM 488 Protein synthesis Assay Kit (Invitrogen, Carlsbad, CA, USA) as described previously, with a slight modification [34]. To label newly synthesized proteins at the mitoribosome, cytosolic translation activity was inhibited by preincubating cells with 25 μg/mL emetine (Merck Millipore, Burlington, MA, USA) for 2 h. Then, cells were incubated in methionine-free RPMI medium (Invitrogen) supplemented with 50 μM L-homopropargylglycine (Click-iTTM HPG, Invitrogen) for 1 h. After being washed twice with PBS, the cells were lysed in lysis buffer (1% SDS, 50 mM Tris, pH 8.0, 1 mM NaF, 1 mM Na_3_VO_4_, 1 mM PMSF, 1.5 μg/mL pepstatin A, 1.5 μg/mL leupeptin). Whole-cell lysate (50 μg) was further conjugated with Alexa Fluor 488 in a Click-iTTM reaction cocktail for 30 min according to the manufacturer’s protocol and subjected to Western blot analysis using Alexa Fluor 488 antibody (A-11094, Invitrogen). The specificity of mitochondrial translation activity was confirmed using chloramphenicol (Sigma, St. Louis, MO, USA).

### 2.14. Immunofluorescence–Fluorescence In Situ Hybridization (IF–FISH) Analysis for Telomeric DNA Damage

To visualize telomeric DNA damage, cells grown on coverslips were first subjected to conventional immunofluorescence staining using 4% paraformaldehyde (for fixation), primary 53BP1 antibody (4937S, Cell signaling Technology, Danvers, MA, USA), and TRITC-conjugated anti-rabbit secondary antibody (A16101, Invitrogen). After the staining, the cells were again subjected to FISH staining according to the protocol provided with the FITC-labelled telomere specific (TTAGGG) PNA probe (PANAGENE, Daejeon, Korea). Nuclei were counter-stained with 1 μg/mL DAPI (D3571, Invitrogen, Carlsbad, CA, USA). Fluorescence-stained cells were visualized with a Nikon A1R Spectral Confocal Laser Dual Scanning Microscope (Nikon Instruments Inc., Melville, NY, USA).

### 2.15. Statistical Analyses

Statistical analyses of transcriptomes were performed using R (v4.0.3), as described above, and the other experimental data were evaluated by unpaired two-sample Student’s *t*-tests using GraphPad Prism software (La Jolla, CA, USA). 

## 3. Results

### 3.1. Deregulation of Mitoribosomal Genes Is an Initial Event of Replicative Senescence

To investigate how mitochondrial defects could be involved in the process of replicative senescence (RS), we analyzed the expression profiles of mitochondria-associated genes (MAG) encoding mitochondrial proteins listed in MitoCarta 3.0 [5], using the time-series transcriptomes of RS of HDFs, previously generated by our group [25]. The HDFs undergoing four stages of senescence, previously defined by distinct gene expression patterns [25], early (E), middle (M), advanced (A), and very advanced (VA), were clustered into three groups based on the MAG expression profiles, in which cells at A and VA stages were clustered together (Figure 1A,B). These data imply that the major deregulation of MAGs is completed before the entry into A stage. The fuzzy c-means clustering of RS model using 1113 MAGs identified 10 gene clusters (RS-M1 to M10) with distinct time-dependent expression patterns (see Materials and Methods; Figure 1C). To identify the causative MAGs involved in mitochondrial defects associated with senescence progression, we analyzed three clusters showing progressive downregulation from the E to VA stage of RS, denoted as RS-M1 to M3 (38.0% of 1113 genes; Figure 1D). RS-M1 to M3 clusters mainly consisted of genes categorized as mitochondrial central dogma (MCD) and metabolism according to the MitoCarta3.0, in which 57 genes were found to encode MRPs (Figure 1E). The fuzzy c-means clustering of the RS model using MRP and cytosolic ribosomal proteins (RPs) further identified 10 gene clusters, of which 6 clusters showed downregulated expression patterns over the doubling time (DT) (Supplementary Figure S1; see Materials and Methods). Notably, downregulation of MRPs mostly occurred during the E-to-M transition (88.7%, 47 vs. 53 MRPs) while that of the majority of RPs was observed at later time points (89.4%, 74 vs. 83 RPs) (Figure 1F,G). These results together indicate the occurrence of mitoribosomal defects at the initial E-to-M transition prior to cytosolic ribosomal defects in the RS process of HDF.

### 3.2. Identification of a Senescence-Associated Mitoribosomal Gene Signature

**Figure 2:**
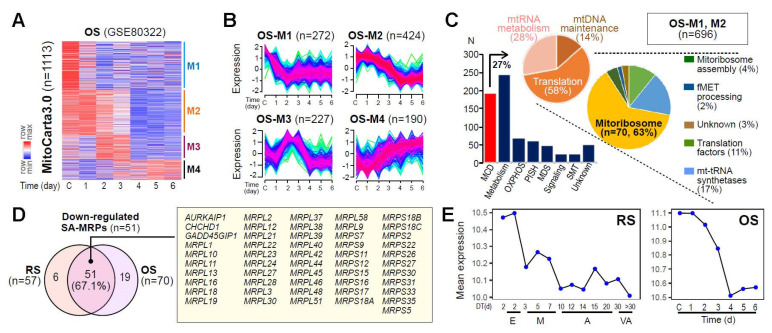
Identification of the SA-MPRs commonly downregulated in cellular senescence. (**A**) Expression heatmap of genes from MitoCarta 3.0 in OS of HDFs. (**B**) Fuzzy c-means clustering of genes from MitoCarta 3.0 identified four gene clusters, termed M1 to M4. (**C**) Bar chart and pie charts showing the number and proportion of genes assigned to each category defined by MitoCarta 3.0. (**D**) Venn diagram showing the number of the identified MRPs with downregulated expression patterns over time in each cellular senescence model. Gene symbols of commonly downregulated MRPs in both models are shown in the yellow-colored box. (**E**) Mean gene expression of commonly downregulated MRPs are shown for RS (left) and OS (right).

To evaluate whether MRP deregulation could represent a common event in cellular senescence, we further analyzed the expression profiles of MAG derived from the time-series transcriptome data for the oxidative stress-induced senescence (OS) model of HDF, previously generated by our group [27]. As observed in the RS model, the fuzzy c-means clustering of MAG identified four distinct gene clusters, of which two clusters (OS-M1 and M2, 62.5% of 1113 genes) showed progressive downregulation over time (Figure 2A,B). Of these downregulated genes, 70 genes encode mitoribosomal proteins according to MitoCarta3.0 (Figure 2C). In the two analyzed cellular senescence models (RS and OS), 51 MRPs (67.1% of 82 total MRPs) were commonly downregulated at the early time points (Figure 2D,E), highlighting the potential involvement of the senescence-associated (SA)-MRP gene signatures in OXPHOS dysfunction and consequential deregulation of mitochondrial bioenergetics. 

### 3.3. Downregulated Protein Expressions of MRPs Are Linked to Early Senescent Phenotypes and Mitochondrial ROS Generation 

**Figure 3:**
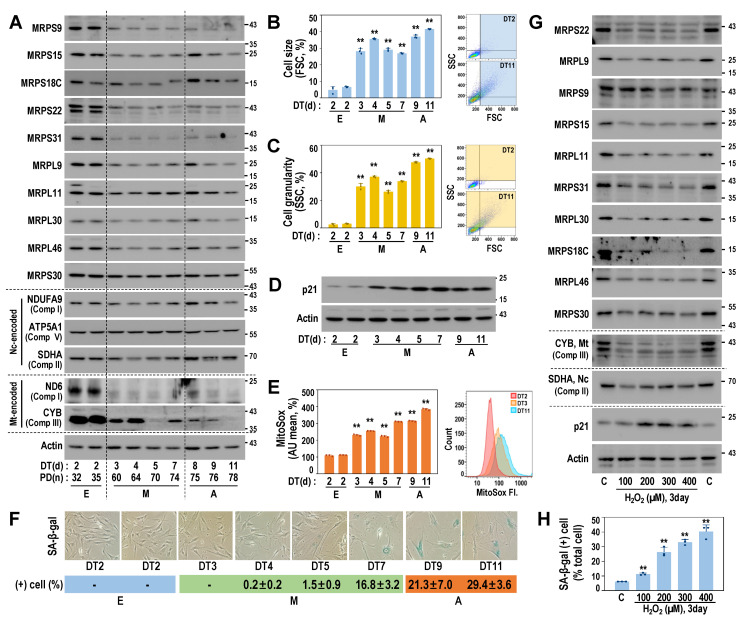
Early MRP downregulation is associated with mitochondrial dysfunction and gain of early senescence phenotypes. (**A**–**F**) RS was developed using primary HDFs, as described in Materials and Methods. (**A**) Time-series Western blot analysis of HDF-RS. Quantifications of three independent experiments are presented in Supplementary Figure S2. Representative blot images are shown. (**B**) Cell size increase in suspended cells was estimated by flowcytometric analysis of forward scattering (FSC). Representative FSC patterns are shown in the right panel. (**C**) Cell granularity increase was estimated by side scattering (SSC) analysis. Representative SSC patterns are shown in the right panel. (**D**) Western blots of time-series RS of primary HDF. (**E**) Mitochondrial ROS levels of the cells were monitored by MitoSox staining followed by flow cytometric analysis. Representative fluorescence shift is shown (right panel). **, *p* < 0.01 vs. DT2 by Student’s *t*-test in (**B**,**C**,**E**). (**F**) SA-β-gal activity. Representative image (upper) and quantification (lower). (**G**,**H**) HDF-OS model was developed by H_2_O_2_ treatment, as described in Materials and Methods. Cellular responses to different doses of H_2_O_2_ are shown. (**G**) Western blots of the HDF-OS model. Quantifications of three independent experiments are presented in Supplementary Figure S3. Representative blot images are shown. (**H**) SA-β-gal activity. **, *p* < 0.01 vs. C by Student’s *t*-test.

To estimate the functional manifestation of the SA-MRP signatures, we next examined the protein expressions of several SA-MRPs using the RS model of HDF. Notably, protein levels of the 10 selected SA-MRPs were decreased from the M stage, accompanied by the suppression of mtDNA-encoded OXPHOS proteins (ND6 and CYB), without the alteration of ncDNA-encoded OXPHOS proteins (NDUFA9, ATP5A1, and SDHA) (Figure 3A and Supplementary Figure S2). These results indicate that the downregulated SA-MRP signature may be closely linked to mitoribosomal translation activity. Concurrently, we observed increases in cell size and cell granularity, as well as the induction of p21 (Figure 3B–D), which represent the early senescent phenotypes, typically acquired prior to the increased activity of senescence-associated β-galactosidase (SA-β-gal), which is the well-established senescence marker (Figure 3F). In addition, the levels of mitochondrial reactive species (ROS) were increased from the M stage (Figure 3E), suggesting the dysfunction of mitochondrial OXPHOS. In line with the findings observed in the RS model, the expression levels of SA-MRP proteins and *medina* encoded protein (CYB) were decreased in the OS model (Figure 3G,H and Supplementary Figure S3). 

### 3.4. Mitoribosomal Perturbation Triggers Cellular Senescence

**Figure 4:**
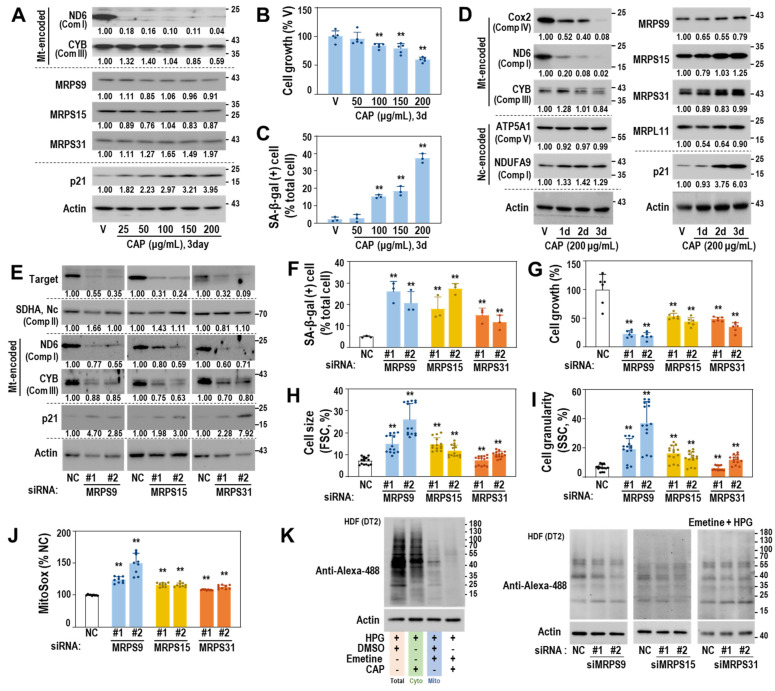
Mitoribosome perturbation induces mitochondrial ROS generation and senescence. (**A**–**D**) HDF (DT2) was treated with the indicated doses of chloramphenicol (CAP), a specific mitoribosome inhibitor. DMSO was used as a vehicle (V) for CAP. (**A**) Western blots. Representative blot images and their quantified values are shown. (**B**) Cell growth. (**C**) SA-β-gal activity. **, *p* < 0.01 vs. V (0.4%) by Student’s *t*-test in (**B**,**C**). (**D**) Western blots. Representative blot images and their quantified values are shown. (**E**–**K**) HDF (DT2) was individually transfected with siRNAs for the indicated MRPs for 4 days. For the negative control (NC), a siRNA with a random sequence was used. (**E**) Western blots. Representative blot images and their quantified values are shown. (**F**) SA-β-gal activity. (**G**) Cell growth. (**H**) Cell size (FSC). (**I**) Cell granularity (SSC). (**J**) Mitochondrial ROS (MitoSOX staining). **, *p* < 0.01 vs. NC by Student’s *t*-test in (**F**–**J**). (**K**) Mitochondrial translation activity. To selectively monitor mitochondrial translation activity, emetine (a cytosolic translation inhibitor) and homopropargylglycine (HPG—a tracer of nascent protein synthesis) were used as described in Materials and Methods. A representative image of protein translation activities for total, cytosol (Cyto), and mitochondria (Mito) is shown in the left panel and selective mitochondrial translation activities after the individual knockdown of the three MRPs are shown in the right panel.

Next, we examined whether functional defects in mitoribosomal translation activity could trigger cellular senescence. The young HDF (DT2) exposed to chloramphenicol (CAP), a selective pharmacological inhibitor of mitochondrial translation [35], showed a notable decrease in protein levels of ND6 and CYB but not in those of MRPs (Figure 4A). In addition, higher concentrations (>100 μg/mL) of CAP significantly induced senescence phenotypes with reduced cell growth, increased SA-β-gal activity, and p21 induction (Figure 4A–C). Further, CAP-induced decrease in the protein expression of mtDNA-encoded proteins (COX2, ND, and CYB) and the induction of p21 were observed over time (Figure 4D). To investigate the effect of genetic suppression of the SA-MRPs, we targeted three small mitoribosomal subunit proteins, MRPS9, MRPS15, and MRPS31, which could effectively block the initiation stage of mitochondrial translation. The siRNA-mediated knockdown of the three MRPs effectively suppressed protein expression of ND6 and CYB (Figure 4E) and significantly induced senescence, as evidenced by increases in SA-β-gal-positive cells, cell size, and granularity, and decrease in cell growth (Figure 4F–I). Importantly, mitochondrial ROS was increased (Figure 4J) and mitochondrial translation activity was reduced (Figure 4K). These data indicate that mitoribosomal deregulation triggers mitochondrial dysfunction and drives a cell into senescence.

### 3.5. Decreased Protein Expression of TPP1 Is an Early Event Driving Cellular Senescence

**Figure 5:**
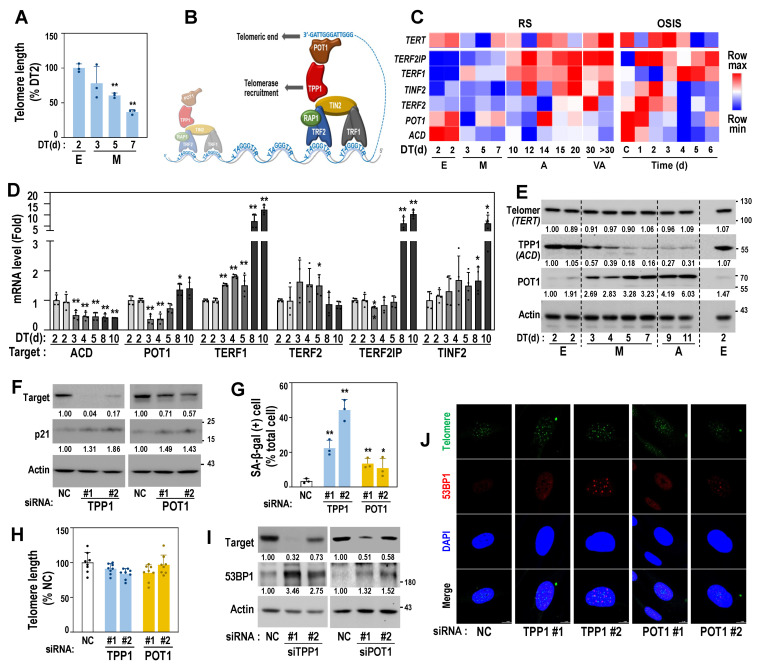
TPP1 expression decreases from the M stage of RS and its suppression induces senescence, accompanied by telomere DNA damage. (**A**) Relative telomere length of HDFs at early (**E**) and middle (M) stages of HDF-RS was estimated, as described in Materials and Methods. **, *p* < 0.01 vs. DT2 by Student’s *t*-test. (**B**) Schematic structure of shelterin complex. (**C**) Expression heatmap of *TERT* (telomerase) and shelterin complex genes in RS and OSIS was obtained from our previous time-series transcriptomic data (GSE41714 and GSE80322). (**D**) Messenger RNA levels of HDFs at the indicated DT of RS model by qPCR analysis. HDFs with two different cell population doubling (PD32 and PD35) were used for DT2. **, *p* < 0.01 and *, *p* < 0.05 vs. DT2 (PD32) by Student’s *t*-test. (**E**) Western blots of time-series HDF-RS. Representative blot images and their quantified values are shown. (**F**–**J**) HDF (DT2) was individually transfected with siRNAs for the target genes for 4 days. (**F**) Western blot. Representative blot images and their quantified values are shown. (**G**) SA-β-gal activity. **, *p* < 0.01 and *, *p* < 0.05 vs. NC by Student’s *t*-test. (**H**) Telomere length analysis. (**I**) Western blot. Representative blot images and their quantified values are shown. (**J**) Telomere dysfunction-induced focus (TIF), a co-localized focus of telomere (green), and 53BP1 (red) were visualized by IF–FISH, as described in Materials and Methods. DAPI (blue) staining was used to visualize nuclei. Quantifications of the TIFs are presented in Supplementary Figure S4A.

To elucidate the association between mitoribosomal deregulation and telomere damage, we monitored telomere length of the cells at E and M stages of RS. Compared to the cells at E stage (DT2), cells at M stage presented shorter telomere length (Figure 5A). Telomeric ends are protected by shelterin complexes, which are composed of six proteins, including TRF1, TRF2, RAP1, POT1, TIN2, and TPP1 (Figure 5B), and their lengths are maintained by telomerase activity regulated by the complexes, depending on cell type (cancer cells and normal cells) [20,21]. The expression profiles of the six shelterin genes derived from the RS transcriptomes showed a notable decrease in the expression of ACD (named TPP1 for its protein) from the M stage onwards with a slight decrease in that of POT1 (Figure 5C, left and 5D). In the OS model, ACD expression was decreased in H_2_O_2_-treated HDFs from day 1 onwards (Figure 5C, right). However, at the protein level, only TPP1, and not POT1, was decreased from the M stage of the RS model (Figure 5E), indicating the functional involvement of TPP1 in the initial entry into RS. Knockdown of TPP1 significantly induced senescence phenotypes, including the gain of SA-β-gal activity and the induction of p21, while POT1 knockdown induced senescence, to a lesser extent (Figure 5F,G). However, the genetic suppression of the shelterin proteins did not substantially affect telomere length (Figure 5H). These results suggest that functional defects in shelterin complexes may induce senescence regardless of genetic origin and without telomere attrition. Next, we further examined how TPP1 suppression induced senescence without telomere attrition. Recently, the involvement of telomere DNA damage in cellular senescence has been demonstrated [22,36]. To monitor DNA damage in telomere regions, we examined the expression of 53BP1, a key player in DNA damage response, and its localization on telomere DNA, known as telomere dysfunction-induced focus (TIF) [37]. The suppression of TPP1 induced increased cellular 53BP1 expression by forming punctate foci within the nucleus (Figure 5I,J) and increased TIFs, of which 53BP1 foci were localized closely to the telomere regions (Figure 5J and Supplementary Figure S4A), indicating the presence of DNA damage at the telomeric end. 

Telomere dysfunction-induced focus (TIF), a co-localized focus of telomere (green), and 53BP1 (red) were visualized by IF–FISH as described in Materials and Methods. DAPI (blue) staining was used to visualize nuclei. Quantifications of the TIFs are presented in Supplementary Figure S4A.

### 3.6. Mitoribosomal Deregulation Functions Upstream of Transcriptional TPP1 Suppression

**Figure 6:**
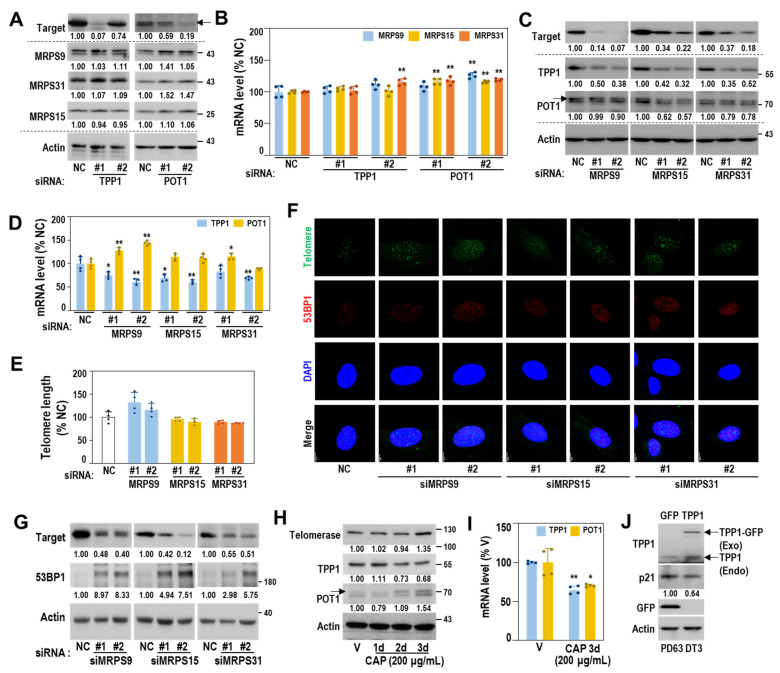
SA-MRP deregulation plays a role as an upstream regulator of TPP1 suppression. (**A**,**B**) HDFs (DT2) were transfected with siRNAs against targets (TPP1 and POT1) for 4 days. (**A**) Western blots. Representative blot images and their quantified values are shown. (**B**) Messenger RNA levels by qPCR 4 days after individual knockdown of TPP1 and POT1. **, *p* < 0.01 vs. NC by Student’s *t*-test. (**C**–**G**) HDFs (DT2) were transfected with siRNAs against target MRPs for 4 days. (**C**) Western blots. Representative blot images and their quantified values are shown. (**D**) Messenger RNA levels (qPCR) 4 days after individual knockdown of MRPS9, MRPS15, and MRPS31. **, *p* < 0.01 and *, *p* < 0.05 vs. NC by Student’s *t*-test. (**E**) Relative telomere length. (**F**) Telomere dysfunction-induced focus (TIF), a co-localized focus of telomere (green) and 53BP1 (red) was visualized by IF–FISH, as described in Materials and Methods. DAPI (blue) staining was used to visualize nuclei. Quantifications of the merged images are presented in Supplementary Figure S4B. (**G**) Western blot. Representative blot images and their quantified values are shown. (**H**,**I**) HDF (DT2) was exposed to CAP (200 μg/mL) for the indicated periods. (**H**) Western blots. Representative blot images and their quantified values are shown. (**I**) Quantitative RT-PCR. **, *p* < 0.01 and *, *p* < 0.05 vs. NC by Student’s *t*-test. (**J**) Western blot analysis after HDF (M stage, PD63 and DT3) was transfected with the pCMV6-ACD-AC-GFP plasmid for 2 days. Representative blot images and their quantified values are shown. (**A**,**C**,**H**) arrow indicated POT1 band.

Next, we examined the interrelationship between mitoribosome deregulation and TPP1 suppression. Knockdown of TPP1 or POT1 did not affect mRNA and protein expressions of the three MRPs (MRPS9, MRPS31 and MRPS15) (Figure 6A,B). However, individual knockdown of the three MRPs selectively decreased both mRNA and protein expressions of TPP1 without decreasing POT1 expression and altering telomere length (Figure 6C–E). Increased 53BP1 expression and TIFs were also observed by knockdown of the three MRPs (Figure 6F,G and Supplementary Figure S4B). In addition, CAP-mediated inhibition of mitoribosomal activity decreased the expression levels of TPP1, but not POT1 and telomerase (Figure 6H,I). When TPP1 was overexpressed in HDF of the M stage (PD63, DT3), p21 induction was reduced, implying a delayed senescent process (Figure 6J). These findings indicate that mitoribosomal deregulation plays a role as an upstream regulator of TPP1 suppression-mediated senescence. 

### 3.7. Gene Expressions of MRPs and Shelterin Complex Are Downregulated in Aged Human Cells and Rat and Mouse Skin Tissues

**Figure 7:**
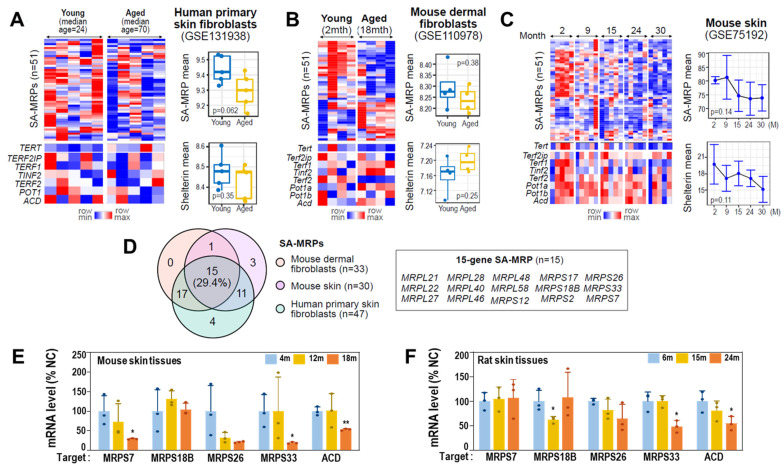
Validation of SA-MRP deregulation in aged human cells and aging mouse/rat skin tissues. (**A**–**C**) Expression heatmaps of SA-MRPs and shelterin genes in human primary (**A**) skin fibroblasts, and mouse (**B**) dermal fibroblasts and (**C**) skin tissues. The mouse genome has two *POT1* orthologs, *Pot1a* and *pot1b*. The Welch *t*-test *p*-values are shown in each right panel. (**D**) Venn diagram showing the number of identified MRPs with downregulated expression patterns in aged samples in each analyzed dataset. Gene symbols of the 15 shared SA-MRPs in all human and mouse cells and tissues employed in this study are shown in the box. (**E**) Messenger RNA levels (qPCR) of target genes using aging mouse skin tissues. **, *p* < 0.01 and *, *p* < 0.05 vs. 4 m by Student’s *t*-test. (**F**) Messenger RNA levels (qPCR) of target genes using aging rat skin tissues. *, *p* < 0.05 vs. 6 m by Student’s *t*-test.

To validate deregulations of MRPs and shelterin complex genes in aged cells and tissues, we next analyzed publicly available transcriptomes of skin tissues and isolated primary cells (see Materials and Methods). Compared to human primary skin fibroblasts from young donors, those from old donors showed decreased expression levels of the 51 SA-MRPs, though they did not reach statistical significance (*p* = 0.062; Figure 7A). Similar results were obtained from mouse primary dermal fibroblasts and skin tissues (Figure 7B,C). Through unsupervised hierarchical clustering of the two analyzed datasets using the expression profiles of 51 SA-MRPs, we identified specific SA-MRPs that were significantly downregulated in aged cells and skin tissues (Supplementary Figure S2), of which 15 SA-MRPs were commonly downregulated in aged human and mouse fibroblasts (Figure 7D). However, expression of shelterin genes did not reach statistical significance, although slightly decreased expression of ACD was only observed in aged human cells (Figure 7A–C). It is noteworthy that TERT was slightly increased in aged human cells, while it was decreased in aged mouse fibroblasts and tissues (Figure 7A–C), indicating differential involvement of telomerase in the two species. In particular, the expression levels of the SA-MRPs and the shelterin genes were progressively decreased with age in mouse skin tissues (Figure 7C). To validate these findings, we performed qRT-PCR using mouse skin tissues obtained at three different ages (4, 12, and 18 months) and found decreased mRNA expression levels of specific SA-MRPs, including Mrps7, Mrps33, and MRPS26, and Acd, in aged specimens (Figure 7E). Similarly, decreased expression levels of Mrps33 and Acd were further confirmed (Figure 7F) using aging rat skin tissues (6, 15, and 24 months).

### 3.8. Single-Cell Transcriptomes of Human Skin Reveal Early Suppressed Expressions of SA-MRPs and Shelterin Genes 

**Figure 8:**
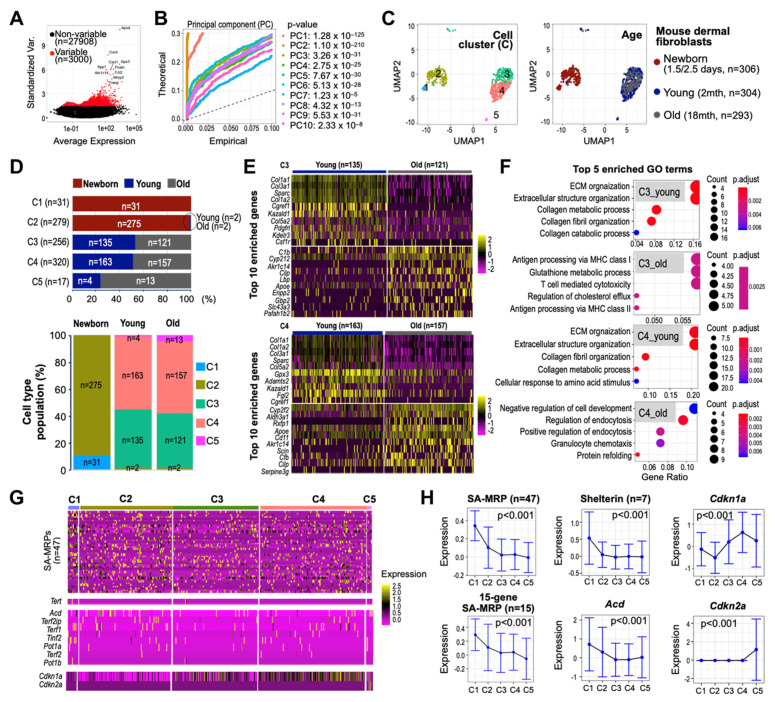
Single-cell RNA-seq analysis identifies distinct cell clusters with unique expression profiles of MRPs and shleterin genes associated with senescence. (**A**) Identification of highly variable features in scRNA-seq data derived from aging mouse dermal fibroblasts (GSE111136). (**B**) Distribution of *p*-values for each PC with a uniform distribution (dashed line) showing significant PCs. (**C**) Uniform manifold approximation and projection (UMAP) plots depicting cells of different Seurat-defined cell clusters (left) and ages (right). (**D**) Stacked bar charts showing the number and proportion of cells assigned to Seurat-defined cell clusters. (**E**) Expression heatmaps of the top 10 genes for each sample (young and old) in C3 (top) and C4 cells (bottom) only. (**F**) Dot plot showing top GO terms enriched in each cell group. (**G**) Expression heatmaps and (**H**) mean expression (±SD) of the 47 mouse SA-MRPs, 15 SA-MRP genes, shelterin complex, Acd, and senescence markers (Cdkn1a for p21 and Cdkn2a for p16). Kruskal–Wallis chi-squared *p*-values (*p*) are shown. *N* refers to the number of genes analyzed. Of the 51 SA-MRPs, *Mrps16*, *Mrps37*, *Mrps30*, and *Mrps27* are missing in the filtered dataset.

To assess cellular heterogeneity of the SA-MRPs and shelterin complex expression and their association, we processed and analyzed scRNA-seq dataset derived from aging dermal fibroblasts (see Materials and Methods). The principal component analysis (PCA) was performed on the scaled data comprising 1044 QC-passed cells and 3000 highly variable features to determine the number of principal components (PCs) for cell clustering (Figure 8A,B). Using the first 10 PCs, murine aging dermal fibroblasts obtained at three different ages (newborn (P1.5–2.5 days), young (2 months old), and old (18 months old)) were clustered into six cell subpopulations, in which one cell cluster (C6) predominantly expressing mitochondrial genes was excluded from further analyses (Supplementary Figure S3). Of the remaining five cell clusters (denoted C1 to C5), cells of C1 and C2, mostly derived from fibroblasts of the newborn mouse, were clearly separated from those of C3 to C5, derived from young and old mice, on the UMAP plot (Figure 8C). The identified cell subpopulations comprised varying proportions of cells from different ages, suggesting heterogenous age-defining characteristics of cell clusters (Figure 8D). We further found that the constituent cell subpopulations of the fibroblasts of the young mouse were comparable to those of the old mouse. The gene ontology (GO) analyses of the top enriched features of C3 and C4 cells identified the extracellular matrix (ECM) organization and extracellular structure organization as top enriched GO terms in the young mouse (Figure 8E,F and Supplementary Figure S4), consistent with recent scRNA-seq findings for human skin from young donors [38]. While PCA data suggest that skin tissues from young and old mice have comparable composition in terms of cell subpopulations, downstream analyses clearly indicate that different cell subpopulations uniquely express age-defining features, highlighting the importance of cluster-determining features present in young and old mice for deciphering fibroblast heterogeneity in aging skin. Importantly, we found a progressive decrease in the expression of MRPs and shelterin complex components, including Acd, in cells of C3 to C5 compared to C1 and C2, with increased expression of p21 and p16 (Figure 8G,H). These results suggest that deregulation of SA-MRPs and Acd occurs before mice reach the age of 2 months, supporting our claim about the potential role of SA-MRP and TPP1 as the regulators of the early stage of senescence.

## 4. Discussion

Senescent cells increasingly accumulate in aged tissues and may further contribute to the development of age-related pathologies [39,40,41]. Emerging preclinical data derived from animal models provide a new potential therapeutic avenue of senolytics, which selectively induces the apoptosis of senescent cells [42,43]. Despite the efficacy of their removal, there is still a lack of robust senescence-specific markers, particularly those that can allow the reliable detection of early stages of senescence. Currently, SA-β-gal represents the most widely used biomarker for senescent cells, but it appears after other senescence phenotypes, such as increases in ROS, cell size, and cell granularity, which arise at later stages of senescence. Moreover, these earlier phenotypes are accompanied by initial gene reprogramming [25], opening a new avenue for developing specific gene markers of the early stages of senescence. Here, through the comprehensive analysis of our transcriptome data for the two senescence models (RS and OS) previously generated by our group, we report the collective MRP deregulation occurring during the E-to-M stage transition prior to the exhibition of noticeable SA-β-gal activity. The 51 SA-MRPs were identified as a key signature for the initial entry into HDF senescence. By employing publicly available transcriptome analyses of aged human primary fibroblasts, mouse dermal fibroblasts, and our newly generated validation data derived from aging mouse and rat skin tissues, we further found that collective MRP deregulation was the common event in the cell senescence and aging process and identified 15 deregulated SA-MRP genes (*MRPL21*, *MRPL22*, *MRPL27*, *MRPL28*, *MRPL40*, *MRPL46*, *MRPL48*, *MRPL58*, *MRPS12*, *MRPS17*, *MRPS18B*, *MRPS2*, *MRPS26*, *MRPS33*, and *MRPS7*) as the shared signature of MRP deregulation across different species and cell types. 

Mitoribosomes are macrostructures of dual genetic origin, including 2 mtDNA-encoded mitoribosomal RNAs and 82 nuclear DNA-encoded MRPs, which are specialized for translation of the core 13 mtDNA-encoded proteins of OXPHOS governing mitochondrial energetics [12]. We also demonstrated that individual knockdown of several MRPs (MRPS9, MRPS15, and MRPS31) in primary young HDF triggered senescence along with a decrease in mitochondrial translation activity and an increase in mitochondrial ROS. These findings indicate that MRP deregulation-mediated mitochondrial dysfunction may represent an early event promoting the cell’s entry into senescence. In line with our findings, Houthkooper at al. identified several MRPs, including MRPS5, as metabolic and longevity regulators in *C. elegans* [44]. While many studies have reported altered expression levels of MRPs in cancer [45], the detailed molecular mechanisms underlying their involvement in senescence and aging have yet to be elucidated. 

The cellular senescence established by extensive in vitro cell cultures was first described by Hayflick in the 1960s [13]. Since then, the RS of HDF with a finite cellular replicative life span has been widely used for cellular modeling of human aging. The key underlying mechanism of RS has been explained by telomere attrition, or telomere shortening, which is a result of the “end-replication problem” during repetitive cell division [16]. Despite such inevitable telomere attrition induced by the “end-replication problem” in mitotic cells, the repeating telomere DNA sequences have a protective mechanism involving the shelterin complex, which is composed of six proteins (TPP1, POT1, RAP1, TIN2, TRF1, and TRF2). Multiple shelterin complexes bind to telomere DNA sequences to protect the structure via T-loop formation and recruit telomerase enzymes when telomere lengthening is needed [46,47]. TPP1 is known to play an essential function in organizing the shelterin complex by mediating the recruitment of POT1 to telomeres, promoting interaction between TIN2, TRF1, and TRF2 [48,49]. Consequently, TPP1 recruits and regulates telomerase activity for telomere protection [50,51,52]. Conditional knockout experiments demonstrated the role of TPP1 in protecting telomeres through POT1 recruitment [53]. In this study, we found that telomere attrition occurred during the E-to-M transition of the RS progression, which was minimally, and progressively, amplified during the M phase. The mRNA and protein levels of TPP1 were clearly suppressed at the E-to-M transition and were consistent afterwards, implying that the M stage shelterin complexes had limited capacity for telomerase recruitment, despite unchanged telomerase levels. We further demonstrated that TPP1 suppression induced senescence, accompanied by telomere DNA damage, with no significant change in telomere length, and that MRP deregulation functioned as an upstream suppressor of TPP1 expression. However, it remains unclear how MRP deregulation modulates TPP1 expression. One possible explanation is that ROS may play a role as mediators between the two events. This could be derived from our findings as follows: one possibility is that suppression of several MRPs augmented mitochondrial ROS; another is that the OSIS model, with H_2_O_2_ treatment, displayed downregulation of *ACD*, the TPP1 gene. Further detailed studies are required to elucidate the molecular mechanisms relevant to this issue.

Our data suggest that in the RS model the two events of telomere attrition associated with the “end-replication problem” and telomere DNA damage by TPP1 suppression could be developed at the M stage, in which population doubling (PD) number is over 60 (Figure 1A and Figure 3A), and that TPP1-mediated telomere DNA damage alone could trigger senescence without telomere attrition. We posit that the absence of telomere attrition in TPP1 suppression-induced senescence might be attributed to the short experimental period (4 days) for making the “end-replication problem.” Previously, Guo et al. reported that reduced TPP1 expression induced a senescence phenotype in primary mouse fibroblasts [54]. In addition, a recent study demonstrated that cigarette smoke-induced TPP1 reduction caused telomeric DNA damage and lung cell senescence [55], supporting the role of TPP1 in senescence.

While our in vitro data from primary HDF demonstrate that MRP deregulation could drive the early entry into senescence, they were not sufficient to explain how this cellular feature could be involved in tissular and organismal aging progression due to cellular heterogeneity at the senescent stage, even of single-cell type, in organismal tissues. To address this, we analyzed a publicly available scRNA-seq dataset derived from aging mouse dermal fibroblasts and observed the existence of five different cell subpopulations (C1, C2, C3, C4, and C5) and varying composition of these subpopulations in mice of different age, suggesting heterogenous age-defining characteristics of cell subpopulations. Importantly, we found that the expression levels of MRPs and shelterin genes were progressively decreased from C1 to C5, in which C1 and C2 cells were derived mostly from newborn mice (1.5/2.5 days old) while comparable numbers of C3 and C4 cells were obtained from young (2-month-old) and old (18-month-old) mice. We speculate that MRPs and shelterin genes, including *Acd*, are suppressed between newborn and young ages. We could not, however, pinpoint the exact aging time point at which the MRPs and shelterin genes are suppressed, as the analyzed dataset was derived from only three different aged mice (newborn (P1.5–2.5 days old), young (2 months old), and old (18 months old)). Moreover, there was an observable difference in telomerase expression profile between human primary cells (increase with aging) and mouse primary cells (decrease with aging), suggesting its differential involvement in the aging progress. Regardless, these single-cell analyses support our hypothesis that deregulation of MRPs and TPP1 is the early regulator and indicator of senescence and aging progress. Collectively, we report that MRP deregulation driving TPP1 suppression is the initial entry into human cell senescence, providing the 15 SA-MRPs and TPP1 as novel markers of early senescence, which could not be characterized by SA-β-gal activity. Finally, we uncover the functional significance of TPP1-mediated telomere deprotection in senescence induction.

## Data Availability

The bulk and single-cell RNA seq datasets analyzed in this study can be found at the NCBI GEO under the accession codes GSE41714, GSE80322, GSE75192, GSE110978, and GSE131938.

## Supplementary Materials

The following supporting information can be downloaded at: https://www.mdpi.com/article/10.3390/cells11132079/s1, Supplementary Figure S1: Fuzzy c-means clustering of RS model using MRP and cytosolic RPs identified 10 gene clusters. Clusters showing down-regulated expression patterns over DT during the E-to-M transition are highlighted in red. Supplementary Figure S2: Quantification of time-series MRPs expression of HDF-RS model. Quantified values of target protein levels on the immuno-blots (Figure 3A) were obtained by densitometric analysis using Image J. The value of actin for each blot was used as a control. Heatmap was generated using mean values of MRPs (upper panel) and mitochondrial protein expression (lower). The values are presented as mean ± standard deviation (SD) (*n* = 3). *, *p* < 0.05; **, *p* < 0.01 versus E (mean of two DT2 values) by student t-test. Supplementary Figure S3: Quantification of MRPs expression in dose- responses of HDF-OSIS model. Quantified values of target protein levels on the immuno-blots (Figure 3G) were obtained by densitometric analysis using Image J. The value of actin for each blot was used as a control. Heatmap was generated using mean values of MRPs (upper panel) and mitochondrial protein expression (lower). The values are presented as mean ± standard deviation (SD) (*n* = 3). *, *p* < 0.05; **, *p* < 0.01 versus C by student t-test. Supplementary Figure S4: Quantification of TIFs after knockdown of target genes. TIF quantification was obtained from IF-FISH results of Figure 5J (A) and Figure 6F (B). TIF positive (+) cell were selected for the cell that have at least three TIFs in its nucleus. Values of TIF (+) cells were obtained by counting over 20 cells from five images of three independent cover slips. *p* < 0.01 vs. NC by student t-test. Supplementary Figure S5: Unsupervised hierarchical clustering identified down-regulated SA-MRPs in aged specimens. Expression heatmaps of SA-MRPs (*n* = 51) using publicly available datasets derived from aging human primary (A) skin fibroblasts (GSE131938), and mouse (B) dermal fibroblasts (GSE110978) and (C) skin tissues (GSE75192). One minus pearson correlation and average method were used for metric and linkage clustering, respectively. The Welch t-test *p*-values (A and B) and the Kruskal-Wallis chi-squared p-values (C) are shown (bottom). Supplementary Figure S6. Single-cell RNA-seq analysis identifies six distinct cell clusters with unique expression profiles of MRPs and shleterin genes associated with senescence. (A) Uniform manifold approximation and projection (UMAP) plots depicting cells of different replicate (left), Seurat-defined cell cluster (middle) and age (right). (B) Stacked bar charts showing the number and proportion of cells assigned to Seurat-defined cell cluster. Expression heatmaps of (C) the top enriched genes for each Seurat-defined cell cluster and (D) SA-MRPs and genes encoding shelterin complex. (E) Mean expression (±SD) of the 47 SA-MRPs and shelterin complex genes. Of the 51 SA-MRPs, Mrps16, Mrps37, Mrps30 and Mrps27 are missing in the filtered dataset. Supplementary Figure S7. Expression heatmaps of the top 100 enriched genes for each age group (young and old) in cells of Seurat-defined cell cluster (A) C3 and (B) C4. Dot plots showing top 10 enriched GO terms for each age group in cells of Seurat-defined cell cluster (C) C3 and (D) C4. Supplementary Table S1. qPCR Primer list.
