# Supplementary material for: Mitoribosomal Deregulation Drives Senescence via TPP1-Mediated Telomere Deprotection
Source: Cells. 2022 Jun 30;11(13):2079. doi: 10.3390/cells11132079 (PMC9265344; doi:10.3390/cells11132079)
Supplement: Supplementary file 1 [file cells-11-02079-s001.zip › Supplementary Material.pdf]

Supplementary Material

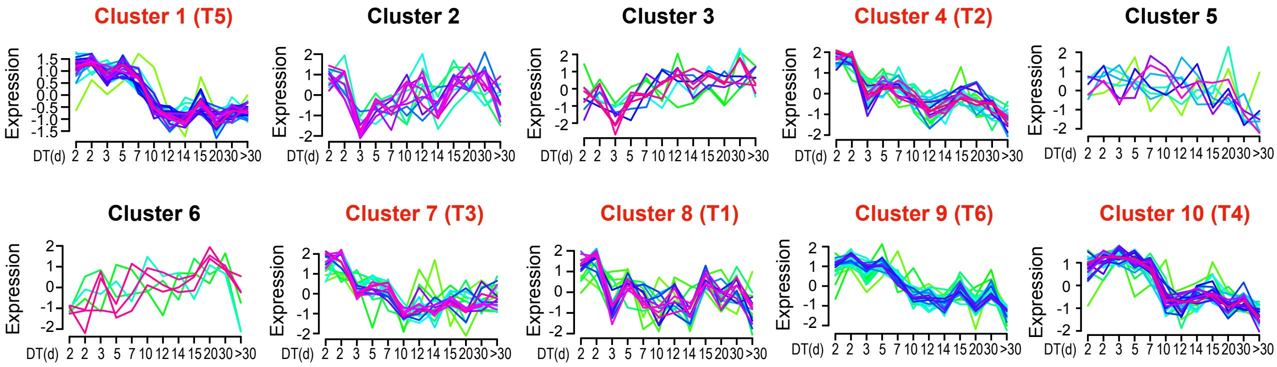

Supplementary Figure S1. Fuzzy c-means clustering of RS model using MRP and cytosolic RPs identified 10 gene clusters. Clusters showing down-regulated expression patterns over DT during the E-to-M transition are highlighted in red.

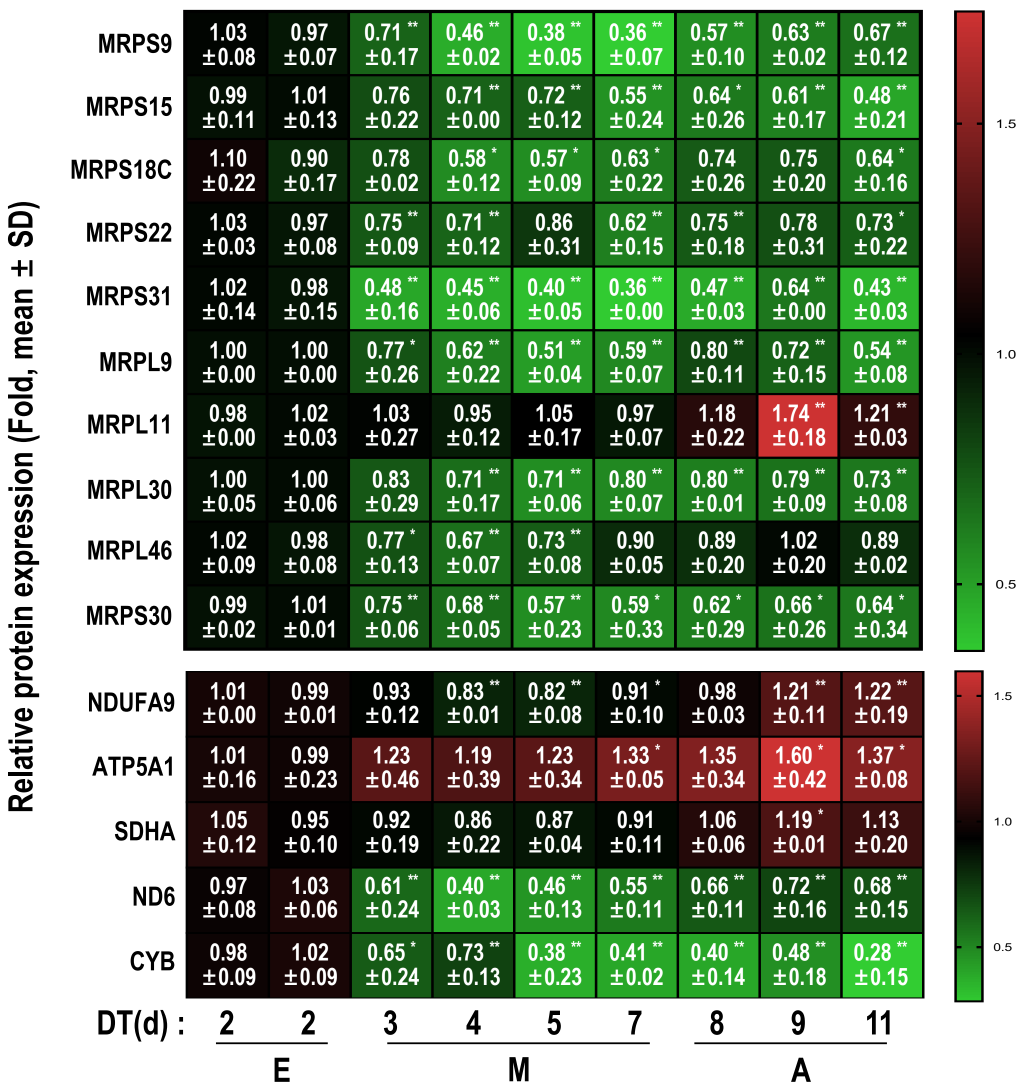

Supplementary Figure S2. Quantification of time-series MRPs expression of HDF-RS model. Quantified values of target protein levels on the immuno-blots (Figure 3A) were obtained by densitometric analysis using Image J. The value of actin for each blot was used as a control. Heatmap was generated using mean values of MRPs (upper panel) and mitochondrial protein expression (lower). The values are presented as mean ± standard deviation (SD) (n=3). *, $p<0.05$; **, $p<0.01$ versus E (mean of two DT2 values) by student t-test.

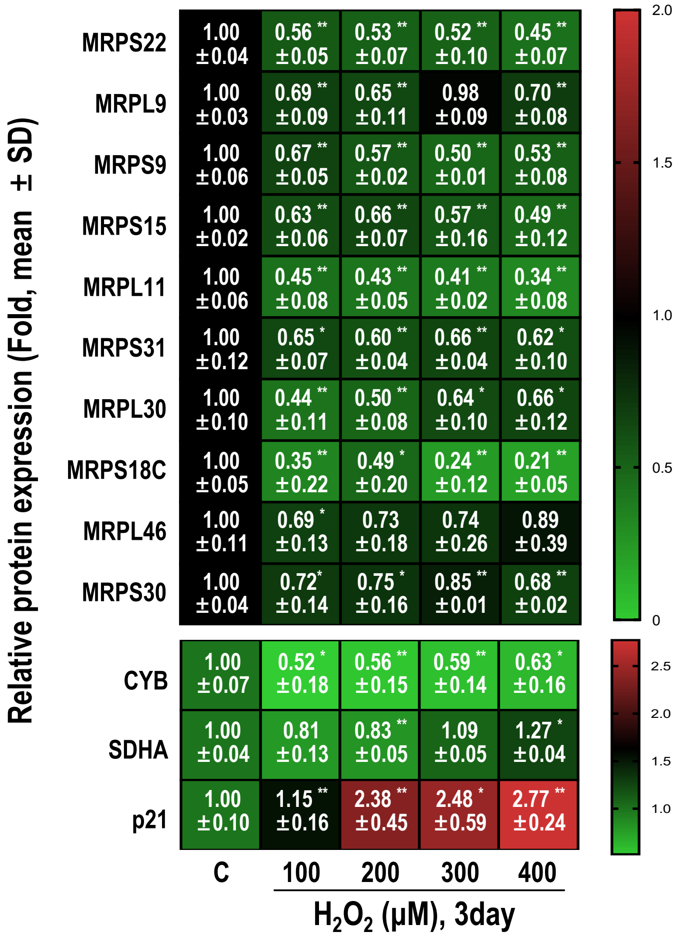

Supplementary Figure S3. Quantification of MRPs expression in dose- responses of HDF-OSIS model. Quantified values of target protein levels on the immuno-blots (Figure 3G) were obtained by densitometric analysis using Image J. The value of actin for each blot was used as a control. Heatmap was generated using mean values of MRPs (upper panel) and mitochondrial protein expression (lower). The values are presented as mean ± standard deviation (SD) (n=3). *, p<0.05; **, p<0.01 versus C by student t-test.

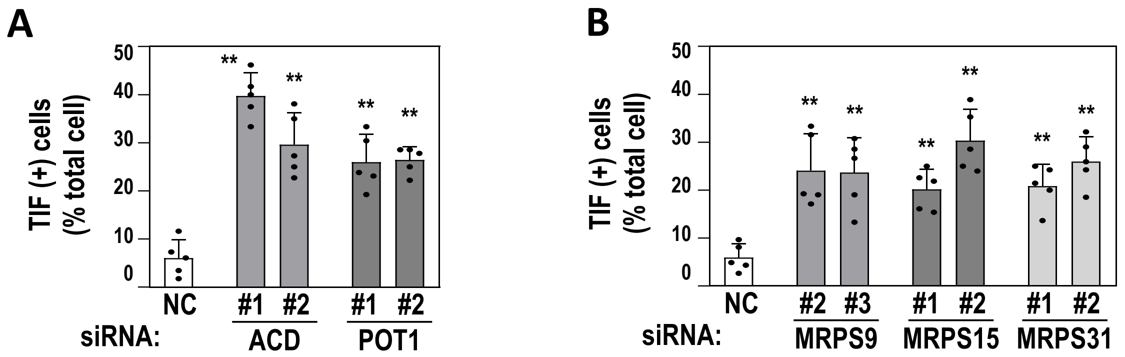

Supplementary Figure S4. Quantification of TIFs after knockdown of target genes. TIF quantification was obtained from IF-FISH results of Figure 5J (A) and Figure 6F (B). TIF positive (+) cell were selected for the cell that have at least three TIFs in its nucleus. Values of TIF (+) cells were obtained by counting over 20 cells from five images of three independent cover slips. p<0.01 vs. NC by student t-test.

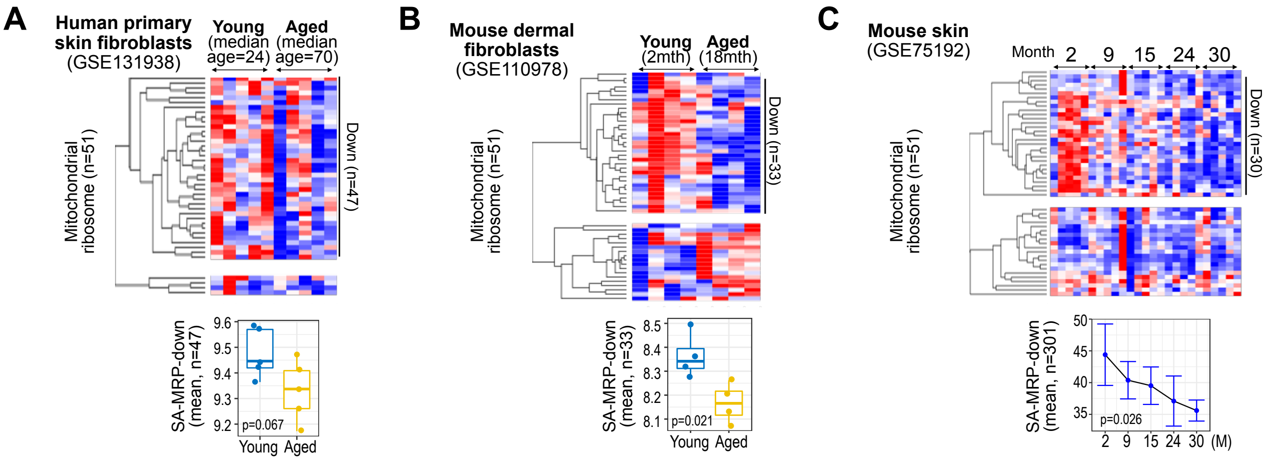

Supplementary Figure S5. Unsupervised hierarchical clustering identified down-regulated SA-MRPs in aged specimens. Expression heatmaps of SA-MRPs (n=51) using publicly available datasets derived from aging human primary (A) skin fibroblasts (GSE131938), and mouse (B) dermal fibroblasts (GSE110978) and (C) skin tissues (GSE75192). One minus pearson correlation and average method were used for metric and linkage clustering, respectively. The Welch t-test p-values (A and B) and the Kruskal-Wallis chi-squared p-values (C) are shown (bottom).

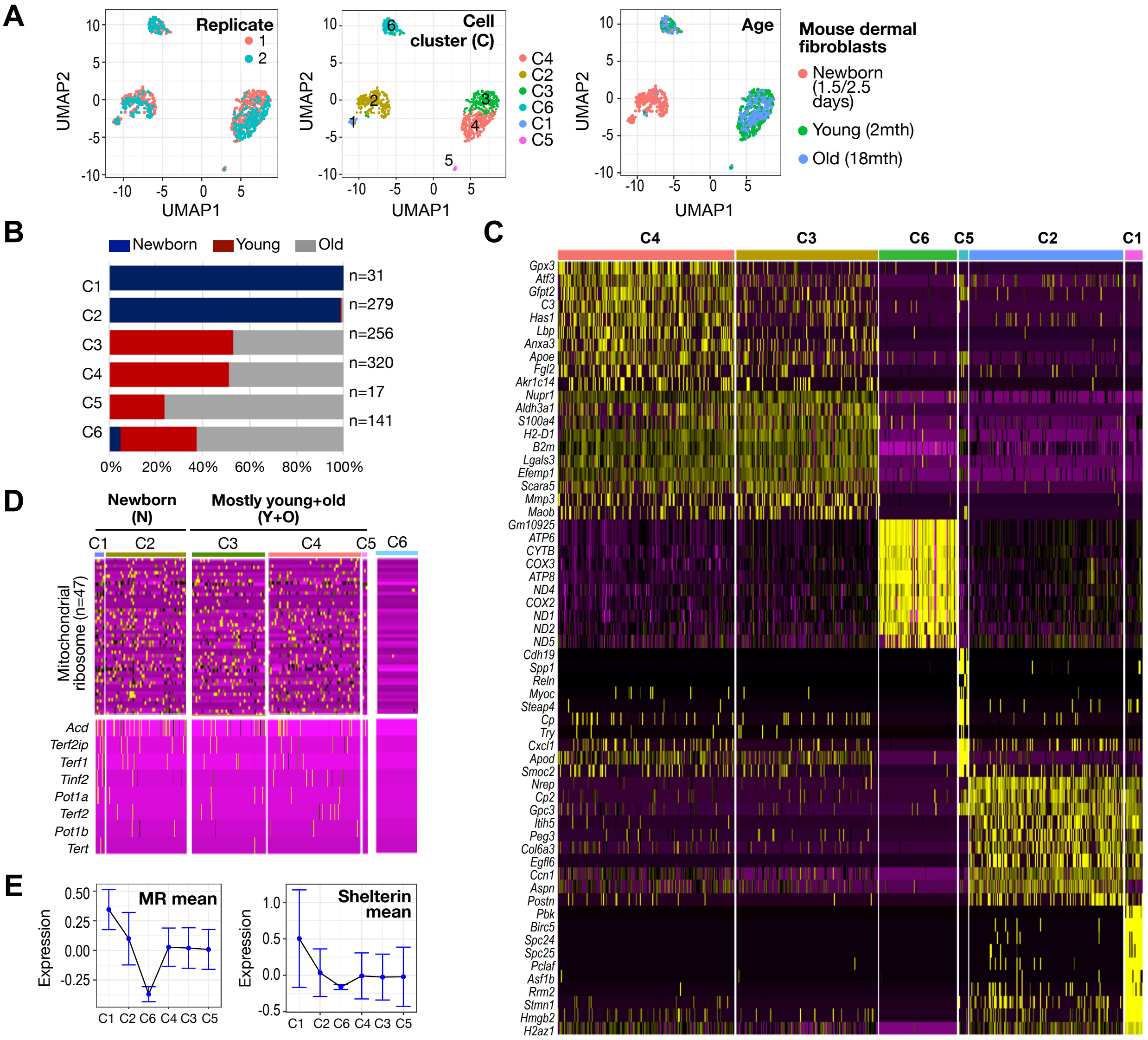

Supplementary Figure S6. Single-cell RNA-seq analysis identifies six distinct cell clusters with unique expression profiles of MRPs and shleterin genes associated with senescence. (A) Uniform manifold approximation and projection (UMAP) plots depicting cells of different replicate (left), Seurat-defined cell cluster (middle) and age (right). (B) Stacked bar charts showing the number and proportion of cells assigned to Seurat-defined cell cluster. Expression heatmaps of (C) the top enriched genes for each Seurat-defined cell cluster and (D) SA-MRPs and genes encoding shelterin complex. (E) Mean expression (±SD) of the 47 SA-MRPs and shelterin complex genes. Of the 51 SA-MRPs, *Mrps16*, *Mrps37*, *Mrps30* and *Mrps27* are missing in the filtered dataset.

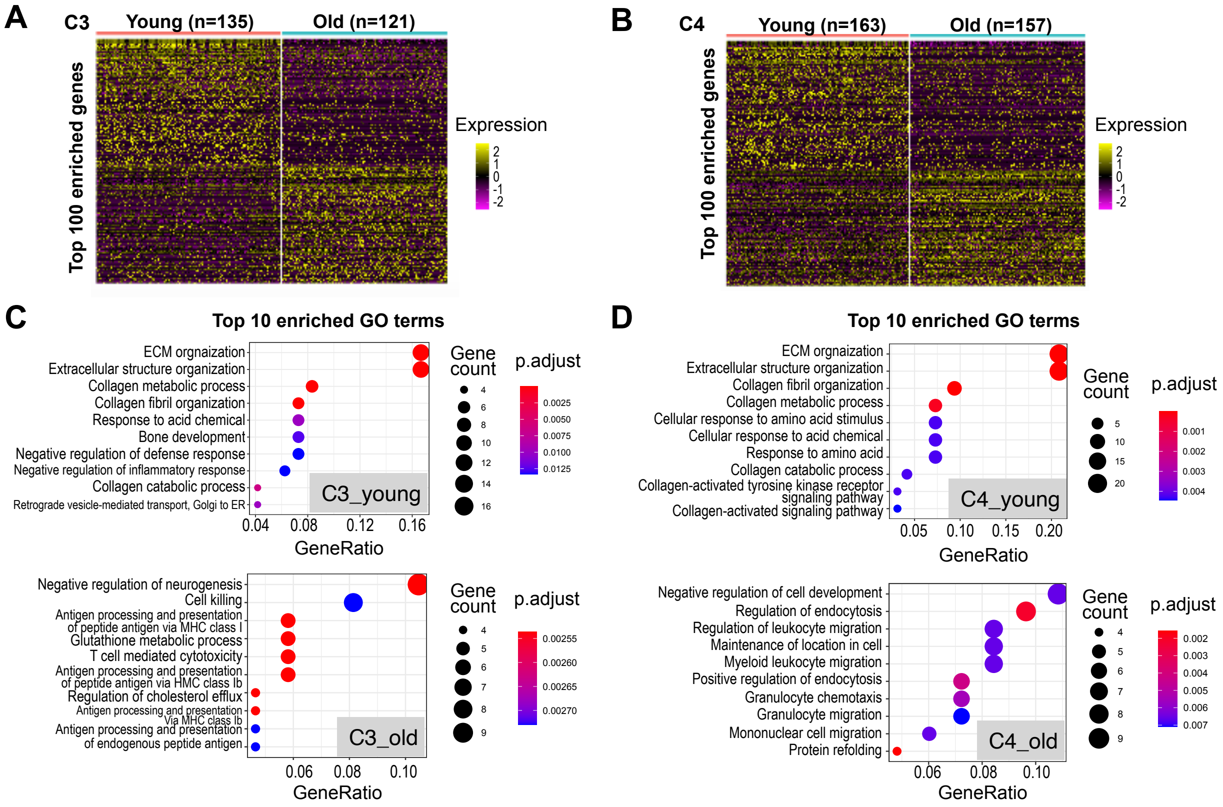

Supplementary Figure S7. Expression heatmaps of the top 100 enriched genes for each age group (young and old) in cells of Seurat-defined cell cluster (A) C3 and (B) C4. Dot plots showing top 10 enriched GO terms for each age group in cells of Seurat-defined cell cluster (C) C3 and (D) C4.
